# Glycated Hemoglobin Levels in Patients with Decompensated Cirrhosis

**DOI:** 10.1155/2016/8390210

**Published:** 2016-11-02

**Authors:** Jeffrey Nadelson, Sanjaya K. Satapathy, Satheesh Nair

**Affiliations:** ^1^Division of Gastroenterology and Hepatology, University of Tennessee Health Sciences Center, Memphis, TN, USA; ^2^Methodist Transplant Institute, Division of Surgery, University of Tennessee Health Sciences Center, Memphis, TN, USA

## Abstract

*Introduction*. Aim of this study is to determine if HbA1c levels are a reliable predictor of glycemic control in patients with decompensated cirrhosis.* Methods*. 200 unique patients referred for liver transplantation at University of Tennessee/Methodist University Transplant Institute with a HbA1c result were included. Three glucose levels prior to the “measured” A1c (MA1c) were input into an HbA1c calculator from the American Diabetes Association website to determine the “calculated” A1c (CA1c). The differences between MA1c and CA1c levels were computed. Patients were divided into three groups: group A, difference of <0.5; group B, 0.51–1.5; and group C, >1.5.* Results*. 97 (49%) patients had hemoglobin A1c of less than 5%. Discordance between calculated and measured HbA1c of >0.5% was seen in 47% (*n* = 94). Higher level of discordance of greater than >1.5 was in 12% of patients (*n* = 24). Hemoglobin was an independent predictor for higher discordance (odds ratio 0.77 95%, CI 0.60–0.99, and *p* value 0.04). HbA1c was an independent predictor of occurrence of HCC (OR 2.69 955, CI 1.38–5.43, and *p* value 0.008).* Conclusion*. HbA1c is not a reliable predictor of glycemic control in patients with decompensated cirrhosis, especially in those with severe anemia.

## 1. Introduction

Hemoglobin A1c (HbA1c) is the gold standard for the measurement of long-range glycemic control in patients with diabetes mellitus. Many studies have reported diabetes mellitus to be a risk factor in patients with alcoholic liver disease and nonalcoholic fatty liver disease (NAFLD) for the development of fibrosis and fibrosis progression [1–3]. In the United States, 2% of adult Americans (5.3 million) are infected with hepatitis B or C [4] and an estimated 31% or more with NAFLD [5, 6].

The presence of CLD is associated with significant impairment in glucose homeostasis. Glucose intolerance is seen in up to 80% of patients with CLD, and frank diabetes is present in 30–60% [7, 8]. Depending on its etiology, CLD has a significant impact on hepatic glucose metabolism.

Among patients with CLD, anemia, portal hypertension, hypersplenism, and variceal bleeding can be common complications. These factors can contribute to longer or shorter red blood cell (RBC) survival [9] and can lead to alteration of the HbA1c. Factors such as nutritional anemia can lead to increased RBC survival and falsely elevated HbA1c levels, whereas bleeding and hemolysis can lower RBC survival time and falsely lower HbA1c values.

Very few studies are dedicated to systematically evaluating the accuracy of HbA1c in CLD patients. In a screening of 20,000 measurements of HbA1c, patients with a very low HbA1c were analyzed further. Of nine abnormally low results, six were the result of cirrhosis, two resulted from hematological neoplasms with anemia, and one was due to hemoglobin F [10]. In a small case series (15 patients with compensated cirrhosis and 20 patients with chronic hepatitis), 40% of cirrhotic patients with diabetes had HbA1c levels below reference range for patients without diabetes [11].

Methodist University Hospital Transplant Institute has been systematically screening its liver transplantation candidates for diabetes with HbA1c levels. Measuring HbA1c is the gold standard for monitoring blood glucose control in diabetes and can now also be used to diagnose diabetes when its results are ≥6.5% [12].

The study objective is to look at glucose and HbA1c levels in cirrhotic patients and determine if HbA1c levels are a reliable predictor of glycemic control in patients who are presented for liver transplantation at Methodist University Hospital Transplant Institute between January 1, 2006, and January 1, 2014.

## 2. Methods

The University of Tennessee Health Sciences Center institutional review board approved this study. The study population included 200 electronic medical records (EMR) of patients who were evaluated for liver transplantation at the Methodist University Hospital Transplant Institute in Memphis, Tennessee, from July 7, 2010, through September 22, 2011. Case selection was restricted to patients who had HbA1c levels in the EMR. Patients who did not have HbA1c levels in the EMR were excluded from the study. Patients were referred for liver transplantation after they were evaluated by a primary care physician or gastroenterologist; the referral was indicated for them based on their clinical presentation and results of laboratory and diagnostic tests such as computed tomography (CT) scans or magnetic resonance imaging (MRI) or Model of End Stage Liver Disease (MELD) scores.

The relevant clinicopathologic information was logged into a Microsoft Access database. We reviewed comprehensive history and physical examination at time of referral to the Transplant Institute. The collected data included patients' demographics, cigarette smoking and alcohol history, past medical history, whether or not they were transplanted, MELD at time of time transplant, liver alone or simultaneous liver-kidney transplant, etiology of liver disease, HbA1c level reported from laboratory, and three glucose levels at time of transplant referral. Three glucose levels prior to the “measured” HbA1c (MA1c) were averaged, logged, and input into an HbA1c calculator from the American Diabetes Association website (http://professional.diabetes.org/diapro/glucose_calc) to determine the “calculated” HbA1c (CA1c). The difference between MA1c and CA1c levels was computed and the patients were divided into three groups: group 1, difference of <0.5; group 2, 0.51–1.5; and group 3, >1.5.

All analyses were performed using the software SPSS (SPSS, Inc., Chicago, Illinois). Data is presented as mean and SD or median and range. Variables between the three groups were analyzed by one-way ANOVA and Chi-square tests for ordinal and categorical variables. *t*-test was used for continuous variables and *z* test for comparison of proportions. A *p* value <0.05 was considered statistically significant.

## 3. Results

The medical records of patients who were evaluated for liver transplantation and had HbA1c levels collected at the Methodist University Hospital Transplant Institute in Memphis, Tennessee, from July 7, 2010, through September 22, 2011, were reviewed, yielding a total of 200 cases. The mean age for this cohort was 54 years with a male predominance: 67% were male. Seventy-nine percent were Caucasian. Forty-four percent and 39% of patients had a history of alcohol abuse and smoking history, respectively. Sixty-two patients (31%) in this study were diabetics. Of those 62 diabetic patients, 26 (42%) were on insulin.

Of the 200 patients included in this study, 55.5% (111) underwent liver transplant and of those 111 patients who underwent liver transplant, 10% (11) underwent simultaneous liver and kidney transplant. Average MELD score at time of transplant was 22. Most common etiology of liver disease in our cohort was hepatitis C virus at 41% (82), followed by hepatocellular carcinoma (HCC) 23% (46), alcohol 22% (44), nonalcoholic steatohepatitis (NASH) 16% (32), primary sclerosing cholangitis 6% (12), primary biliary cholangitis 4.5% (9), and hepatitis B virus 4.5% (9). The remaining 14% (28) of patients in this cohort referred to Transplant Institute included patients with cryptogenic cirrhosis, autoimmune hepatitis, alpha-1-antitrypsin deficiency, Wilson's disease, hemochromatosis, polycystic liver disease, cholangiocarcinoma, and neuroendocrine tumor.

**Figure 1:**
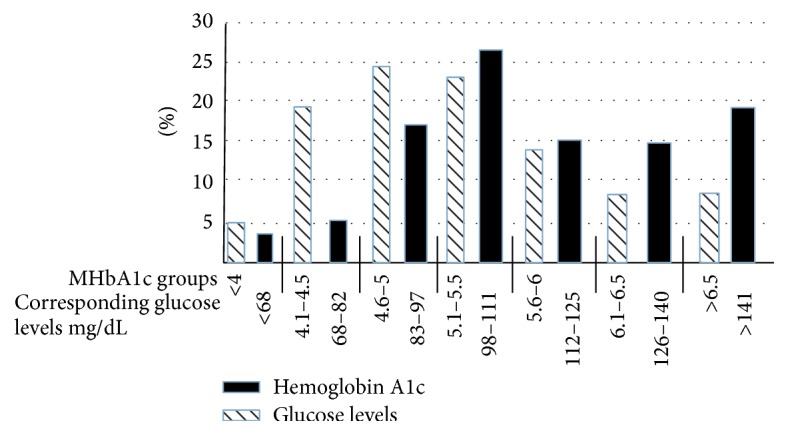
Percentage distribution of HbA1c and average glucose levels in 200 patients. The numbers on *x*-axis represent the measured HbA1c groups and the expected glucose levels (mg/dL) for each group of measured HbA1c.

**Figure 2:**
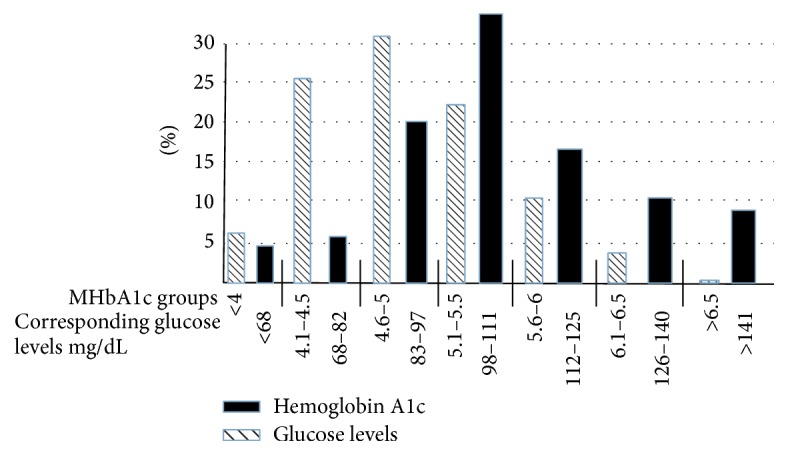
Percentage distribution of HbA1c and average glucose levels in 138 nondiabetic patients. The numbers on *x*-axis represent the measured HbA1c groups and the expected glucose levels (mg/dL) for each group of measured HbA1c.

**Figure 3:**
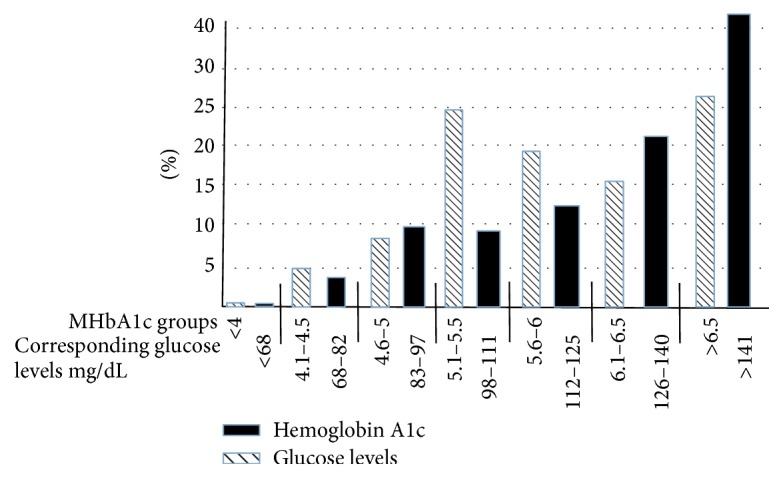
Percentage distribution of HbA1c and average glucose levels in 62 diabetic patients. The numbers on *x*-axis represent the measured HbA1c groups and the expected glucose levels (mg/dL) for each group of measured HbA1c.

The distribution of MA1c and the average glucose levels are depicted in Figure 1 (all patients), Figure 2 (patients without diabetes), and Figure 3 (patients with diabetes). The expected glucose levels for each range of MA1c (*x*-axis) values were obtained from the following website: http://www.diabeteschart.org/bgc1.html. One can infer from the figure that the MA1c values tend to group towards the lower values and actual average glucose values tend to group towards the higher values. For example, among diabetic patients, 58% of patients had MA1c <6, while 77% of patients had actual average glucose levels of more than 140 mg/dL. Similarly 88% of nondiabetic patients had HbA1c <5.5, but 38% had actual average glucose levels of more than 110 mg/dL.

Ninety-seven (49%) patients had HbA1c of less than 5 followed by 72 (36%) patients with hemoglobin A1c of 5-6 and 21 patients (11%) with hemoglobin of more than 7. The discordance between CA1c and MA1c was further categorized into three groups: group 1, the difference between CA1c and MA1c <0.5 (*n* = 106); group 2, the difference between CA1c and MA1c 0.5–1.5 (*n* = 70); and group 3, the difference between CA1c and MA1c >1.5 (*n* = 24). Patients with high discordance were more anemic and had higher incidence of diabetes (Table 1). In a multivariate model including those with high discordance versus those with no discordance, hemoglobin levels were the only independent predictor (odds ratio 0.77 95% CI 0.60–0.99; *p* value 0.04).

Among the 200 patients included in the study, 46 patients had HCC.(29 in nondiabetics (29%) and 17 in diabetics (27%)). As expected, HCC was most commonly seen in patients with hepatitis C and NASH. The clinical features of patients with HCC and without HCC are highlighted in Table 2. In the multivariate model independent predictors of HCC were measured by HbA1c (OR 2.69 955, CI 1.38–5.43, and *p* value 0.008) and age (OR 1.07 955, CI 1.02–1.13, and *p* value 0.004). Female gender was a negative predictor for HCC (OR 0.24 95%, CI 0.94–0.637, and *p* value 0.004). Presence of diabetes was not predictor for HCC.

To further study the relationship between HbA1c and HCC we studied the 62 patients with diabetes. Seventeen patients (27%) had HCC. In a multivariate analysis, HbA1c is an independent predictor of HCC (OR 2.14 95%, CI 1.23–3.70, and *p* value 0.007). There is progressive increase in incidence of HCC with HbA1c levels. Lowest incidence of HCC, 13.4%, is seen in patients with HbA1c being less than 5, followed by 28% for HbA1c levels of 5-6, 38% for patients with HbA1c levels of 6-7, and 50% for patients with HbA1c greater than 7 (*p* value 0.0001).

## 4. Discussion

Cirrhosis and advanced liver disease have been associated with an increased risk for hyperglycemia and diabetes mellitus. The diagnostic yield of common tests used to define diabetes in the general population differs from those with liver disease. HbA1c is a reliable test to assess chronic glycemia and recommended both for the diagnosis and monitoring of diabetes mellitus; however, HbA1c is neither accurate nor reliable in patients with cirrhosis [11]. Various kinds of anemia are common in liver disease, such as macrocytic anemia [13]. Liver cirrhosis promotes glucose intolerance and diabetes through various mechanisms including insulin resistance and impaired insulin secretion. Sixty to 80% of patients with liver disease have glucose intolerance and 10–15% eventually develop overt diabetes [14].

Patients with cirrhosis are frequently anemic and therefore HbA1c measurements may not provide accurate measure of glycemic control as shown in our analysis. Most common causes of anemia in patients with liver disease are chronic gastrointestinal (GI) blood loss from portal hypertension [15–17] and aplastic anemia secondary to viral hepatitis [18–21]. In addition, antiviral therapy, especially ribavirin, for hepatitis C [22–24] or immunosuppressive agents used to treat autoimmune liver diseases can lead to anemia. In patients with alcoholic liver disease, several factors such as nutritional deficiency, malabsorption, and direct toxic effect of alcohol on bone marrow can cause anemia [25–27].

Hypersplenism secondary to portal hypertension is another important mechanism of anemia in patients with chronic liver disease. Hypersplenism is associated with pancytopenia and is almost universally present in patients with cirrhosis. Hemolytic anemia occurs because of intrasplenic destruction of erythrocytes [28]. Any condition that shortens erythrocyte survival or decreases mean erythrocyte age (e.g., recovery from acute blood loss, hemolytic anemia) will falsely lower HbA1c test results [29]. HbA1c results must be interpreted with caution given the pathological processes, including anemia, increased red cell turnover, and transfusion requirements, that adversely impact HbA1c as a marker of long-term glycemic control.

Our cohorts were decompensated cirrhotic patients needing transplant. The etiology of anemia is most likely related to hypersplenism or hemolysis associated end stage liver disease or chronic GI blood loss from portal hypertensive gastropathy. Since etiology of anemia in patients with decompensated cirrhosis is multifactorial, our retrospective study could not analyze the effects of different types of anemia on HbA1c levels in these patients.

Cirrhosis and advanced liver disease have been associated with an increased risk for hyperglycemia and type 2 diabetes mellitus (T2DM). The diagnostic yield of common tests used to define diabetes and insulin resistance in the general population differs significantly from the one observed in patients with liver disease. HbA1c is a reliable test to assess chronic glycemia and recommended both for the diagnosis and monitoring of T2DM; however, HbA1c is neither accurate nor reliable in patients with cirrhosis. A validation study has not been performed to define its true usefulness in the setting of cirrhosis.

The results of our analysis suggest there is discordance in HbA1c measurements in patients with decompensated cirrhosis. The patients with the highest difference between HbA1c levels had significantly more anemia. This study indicates that HbA1c levels are not a reliable predictor of glycemic control in patients with decompensated cirrhosis, especially in those with severe anemia. To date there are limited numbers of studies dedicated to systematically evaluating the accuracy of HbA1c levels in cirrhotic patients. It is critical for providers and patients to have evidence-based data to guide their management strategies to optimize individualized care. Therefore, we conducted a comprehensive review of 200 unique patients referred to the Methodist University Hospital Transplant Institute with at least one HbA1c level to help provide insight and help guide physicians decision-making in this uncertain area.

Although diabetes is a major public health problem and the fifth leading cause of death in the United States [30] it is less known to be associated with increased risks of developing HCC [31–33]. Although such an association could be related to the underlying chronic liver diseases that preceded the development of HCC [34–37], there are several lines of evidence suggesting that diabetes is in fact an independent risk factor for HCC development [37–39]. There is also evidence reporting that findings of HCC recurrence after liver resection and transplantation are seen among patients with diabetes [40, 41]. It is interesting that in our data set diabetes was not independent predictor of HCC, while HbA1c was a strong independent predictor for risk of HCC. Our findings could be extrapolated to indicate that glycemic control could lead to a lower risk of HCC in patients with diabetes.

People with established diabetes have an increased risk of developing certain types of cancers over the general population [42–47]. Previous publications have reported the association of HCC with other comorbidities, such as diabetes mellitus [48]. In our study, Table 2 provides evidence that diabetes can increase the risk of HCC in patients with chronic liver disease. Evidence that diabetes can increase the risk of HCC was also seen in a publication written by Li et al. in 2015 [49]. Hyperinsulinemia, insulin resistance, and cytokine production have been shown to cause fat accumulation in the hepatocytes leading to oxidative stress and progressive liver injury and fibrosis [50–52]. There has also been evidence published showing a relationship between HbA1c levels, elevated liver enzymes, and hepatic steatosis [43]. Recent advances in understanding of NASH and HCC have revealed that HCC to be the leading cause of obesity-related cancer deaths in middle aged men [53].

We conclude that HbA1c levels should only be evaluated in context with all liver function parameters as well as a red blood count in patients with liver disease. Although the pathophysiologic reasons have still not been confirmed, our data demonstrate that HbA1c levels are not a reliable predictor of glycemic control in patients with decompensated cirrhosis, especially in those with severe anemia. This interference may be due to alterations in erythrocyte lifespan and altered protein metabolism, but further investigations are needed to elucidate the exact cause of the interference in patients with liver disease.

## Figures and Tables

**Table 1:** The characteristics of the groups based on difference between the calculated and measured hemoglobin A1c.

	Group 1 (*n* = 106)	Group 2 (*n* = 70)	Group 3 (*n* = 24)
Age	53 ± 11	56 ± 8	52 ± 10
Gender (M/F)	70/36	46/24	18/6
BMI	28.1 ± 5.6	28.6 ± 6.0	28.6 ± 5.8
Etiology of Liver disease (%)			
HCV%/NASH%/alcohol%	40/21/20	40/20/14	50/17/17
DM	26%	31%	50%
Hemoglobin (gm/dL)	12.2 ± 2.3	11.6 ± 1.9	10.9 ± 2.3^*∗*^
Hemoglobin < 10	20%	19%	37%^*∗*^
Hematocrit	36 ± 6.8	34.5 ± 5.6	32.3 ± 7.0^*∗*^
S. creatinine (mg/dL)	1.23 ± 1.3	1.27 ± 1.08	1.23 ± 0.75
Albumin (gm/dL)	3.0 ± 0.7	2.9 ± 0.8	2.9 ± 0.7
Platelets	120 ± 95	122 ± 86	104 ± 71
HBA1c (measured)	5.1 ± 0.7	5.1 ± 1.1	6.0 ± 1.8^*∗*^
Calculated HBA1c#	5.2 ± 0.6	6.0 ± 1.13	8.6 ± 2.6^*∗*^

Group 1: the difference between calculated and measured A1c < 1.0, group 2: the difference between calculated and measured A1c 0.5–1.0, group 3: the difference between calculated and measured A1c > 1.5.

^*∗*^Indicate a *p* value < 0.05.

**Table 2:** Patients with HCC and patients without HCC.

	No HCC (*n* = 154)	with HCC (*n* = 46)	*p* value
Age	52 ± 10	58 ± 6	0.0001
Gender (% males)	61%	84%	0.0001
BMI	28 ± 5.5	28 ± 5.9	NS
Etiology of liver disease			
HCV/NASH/alcohol	36/20/20	59/24/11	0.001
DM	26%	43%	0.01
Hemoglobin	11.5 ± 2.1	13.0 ± 2.1	0.001
Creatinine	1.35 ± 1.3	0.9 ± 0.3	0.029
Albumin	2.9 ± 0.7	3.1 ± 0.8	NS
Platelets	124 ± 98	102 ± 70	NS
HBA1c (measured)	5.1 ± 0.8	5.4 ± 1.6	0.002
